# Th17-Inducing Cytokines IL-6 and IL-23 Are Crucial for Granuloma Formation during Experimental Paracoccidioidomycosis

**DOI:** 10.3389/fimmu.2017.00949

**Published:** 2017-08-21

**Authors:** Fabrine Sales Massafera Tristão, Fernanda Agostini Rocha, Daniela Carlos, Natália Ketelut-Carneiro, Camila Oliveira Silva Souza, Cristiane Maria Milanezi, João Santana Silva

**Affiliations:** ^1^Department of Biochemistry and Immunology, School of Medicine of Ribeirão Preto, University of São Paulo, Ribeirão Preto, Brazil

**Keywords:** paracoccidioidomycosis, Th17, IL-17A, IL-6, IL-23

## Abstract

Paracoccidioidomycosis (PCM), a chronic granulomatous disease caused by the thermally dimorphic fungus *Paracoccidioides brasiliensis* and *Paracoccidioides lutzii*, has the highest mortality rate among systemic mycosis. The T helper 1-mediated immunity is primarily responsible for acquired resistance during *P. brasiliensis* infection, while susceptibility is associated with Th2 occurrence. Th17 is a population of T CD4^+^ cells that, among several chemokines and cytokines, produces IL-17A and requires the presence of IL-1, IL-6, and TGF-β for differentiation in mice and IL-23 for its maintenance. Th17 has been described as an arm of the immune system that enhances host protection against several bacterial and fungal infections, as *Pneumocystis carinii* and *Candida albicans*. In this study, we aimed to evaluate the Th17 immune response and the role of Th17-associated cytokines (IL-6, IL-23, and IL-17A) during experimental PCM. First, we observed that *P. brasiliensis* infection [virulent yeast strain 18 of *P. brasiliensis* (Pb18)] increased the IL-17A production *in vitro* and all the evaluated Th17-associated cytokines in the lung tissue from C57BL/6 wild-type mice. In addition, the deficiency of IL-6, IL-23, or IL-17 receptor A (IL-17RA) impaired the compact granuloma formation and conferred susceptibility during infection, associated with reduced tumor necrosis factor-α, IFN-γ, and inducible nitric oxide synthase enzyme expression. Our data suggest that IL-6 production by bone marrow-derived macrophages (BMDMs) is important to promote the Th17 differentiation during Pb18 infection. In accordance, the adoptive transfer of BMDMs from C57BL/6 to infected IL-6^−/−^ or IL-17RA^−/−^ mice reduced the fungal burden in the lungs compared to nontransferred mice and reestablished the pulmonary granuloma formation. Taken together, these results suggest that Th17-associated cytokines are involved in the modulation of immune response and granuloma formation during experimental PCM.

## Introduction

Paracoccidioidomycosis (PCM), a systemic mycosis characterized by chronic and granulomatous inflammation, is caused by the thermally dimorphic fungus *Paracoccidioides brasiliensis* and *Paracoccidioides lutzii* (1). PCM has high incidence in several countries of Latin America and represents the eighth cause of death among infectious and parasitic diseases. Moreover, it induces the highest mortality rate (1.45 per million inhabitants) among systemic mycosis, being considered an occupational disease and a serious social problem (2). It affects mainly farm workers and usually leads to the formation of pulmonary dysfunction as a consequence of primary infection progression or reactivation of a latent focus (3).

The infection is acquired after inhalation of fungal propagules that convert into the invasive yeast form once in the lungs (4). The resistance to infection is associated with a potent T helper 1 (Th1) response, which induces activation of macrophages that actively control the fungal growth (5, 6). Otherwise, resistance is associated with a predominantly Th2 and Th9 response, inducing eosinophilia and humoral immune responses (7).

Besides the mentioned Th subtypes, classified by defined phenotypic characteristics and specialized functions in immunity, T cells can also differentiate into Th17, a distinct cell lineage that produces various chemokines and cytokines, such as tumor necrosis factor (TNF)-α, IL-6, IL-21, IL-22, IL-17F, and IL-17A (8, 9). IL-1 signaling in T cells is required for the early Th17 differentiation *in vitro* and *in vivo*, and after polarization, IL-1 also allowed Th17 cells to maintain their cytokine secretion profile (10–12). The murine differentiation of Th17 requires the presence of IL-6, IL-21, and TGF-β, which activate Stat3 and induce the expression of the transcription factor retinoic acid-related orphan receptor (RORγt) (10, 13–15). Although IL-23 is not necessary for Th17 differentiation, it is essential for the maintenance of the differentiated Th17 cells (16).

Notably, Th17 cells are described as an arm of the immune system that enhances host protection against several intracellular and extracellular bacterial infections (17). In addition, fungal infections also have been associated with induction of Th17 immune response, triggering effector mechanisms, as production of antimicrobial peptides (8, 18, 19) and factors important for neutrophil function and recruitment (20–22), promoting the resistance to infection (23–25). In fact, the IL-17A and IL-23 production was important in the protective response during experimental *Pneumocystis carinii* infection (26). In addition, *Candida albicans* recognition by antigen-presenting cells promoted the IL-6 and IL-23 secretion, leading to Th17 differentiation and enhancement of host resistance (27–29). Similarly, *P. brasiliensis* was able to induce Th17 and IL-17-producing CD8^+^ T cell (Tc17) differentiation (30, 31).

The cytokine IL-17A coordinates tissue inflammation through higher expression of pro-inflammatory cytokines and chemokines, which collectively determine the magnitude of the inflammatory response. The IL-17A receptors (IL-17RAs) are found in the surface of leukocytes, keratinocytes, fibroblasts, epithelial, mesothelial, and vascular endothelial cells, and its action includes granulopoiesis, neutrophil recruitment, and inflammatory responses (21, 22, 32–34). Moreover, IL-17A plays a critical role in the induction of mature granuloma formation during mycobacterial infection (35). Granulomas are immunological structures important in the host defense against fungi. In the course of granuloma maturation, the recruitment of phagocytes and lymphocytes is triggered by various cytokines and chemokines that are initially produced by infected macrophages, as TNF-α (36). In corroboration, our group showed that TNF-α-deficient mice are highly susceptible to *P. brasiliensis* infection, are not able to mount organized granulomas, and have a great amount of fungus in the lesions and high mortality rates (37). Moreover, IL-17-expressing cells have been detected within and around the granulomas in the skin and oral mucosal lesions from PCM patients (38).

Because little is known about the mechanisms involved in the immune response during PCM, we sought here to investigate the role of IL-6, IL-23, and IL-17A during the experimental *P. brasiliensis* infection. Our data suggest that IL-6-production by bone marrow-derived macrophages (BMDMs) is important to promote the IL-17A production and consequent induction of a protective immune response and granuloma formation during *P. brasiliensis* experimental infection.

## Materials and Methods

### Mice

Male 6- to 7-week-old C57BL/6 mice, and genetically deficient (^−/−^) for IL-6, IL-23p19, and IL-17RA, were obtained from our Isogenic Breeding Unit and maintained under specific pathogen-free conditions in microisolator cages in the animal housing facility of the Department of Biochemistry and Immunology, School of Medicine of Ribeirão Preto, São Paulo University, Ribeirão Preto, Brazil. Mice were supplied with sterilized food and water *ad libitum*. Experiments were conducted according to the ethical principles of animal research adopted by the Brazilian College of Animal Experimentation and approved by the Ethical Commission in Animal Research (protocol 095/2010).

### Fungus, *In Vitro* and *In Vivo* Infections

The virulent yeast strain 18 of *P. brasiliensis* (Pb18) was cultured (7 days at ~36°C) in Brain Heart Infusion (BHI) agar medium (Oxoid Basingstoke, Hampshire, England) supplemented with gentamicin (100 µg/mL) and 5% fetal bovine serum (FBS; Gibco BRL, Life Technologies, Inc.). After, Pb18 colonies were grown for 24 h (37°C, 150 rpm) in F12 Coon’s modified medium (Sigma-Aldrich, St. Louis, MO, USA) containing gentamicin (100 µg/mL). The Pb18 yeast cells were then harvested and washed in sterile phosphate-buffered saline (PBS; pH 7.2), and the fungal viability was determined by fluorescein diacetate-ethidium bromide staining (39). Only high viability suspensions (≥90%) were used in this study. For *in vivo* infection, the concentration of fungal cells was adjusted to 1 × 10^7^ yeasts/mL in PBS and 100 µL of this solution (1 × 10^6^ cells) was intravenously (i.v.) inoculated in each mice. *In vitro* fungal stimulation was performed with a multiplicity of infection (MOI) of 1.

### Splenocyte and Pulmonary Cells Isolation and Activation

The spleen from uninfected male C57BL/6 mice were removed, and single-cell suspensions were obtained after red blood cells lysis (1 min) with buffer containing NH_4_Cl (0.16 M, 9 parts) and Tris-HCl (0.17 M, 1 part). Splenocytes (1 × 10^6^ cells/well) were cultured for 5 days with 1 × 10^6^ Pb18 yeasts cells in RPMI 1640 supplemented with 5% FBS (Gibco), 0.1 mM non-essential amino acids, 1 mM sodium pyruvate, l-glutamine (2 mM), 100 U of penicillin/mL, and 100 µg of streptomycin/mL (all from Sigma-Aldrich). Lung lobules were excised from uninfected and Pb18-infected mice, washed in PBS, minced with scissors, and enzyme digested at 37°C for 30–35 min in 1 mL of digestion buffer [RPMI 1640, 2 mg/mL collagenase IV (Sigma) and 1 mg/mL DNase (Sigma)]. Tissue fragments were further homogenized using a 1-mL pipette, crushed through a 50-µm pore size nylon filter (BD Biosciences, San Jose, CA, USA) and then centrifuged (1,300 rpm, 10 min, 4°C). Next, red blood cells were eliminated using lysis buffer, and remaining cells were washed in PBS, centrifuged, and resuspended in RPMI 1640 containing 5% FBS. For splenocytes and pulmonary cells activation, 1 × 10^6^ cells/well were cultured for 4 h with PMA (50 ng/mL), ionomycin (500 ng/mL), and brefeldin A (5 mg/mL), followed by staining for extracellular and intracellular markers and analysis by flow cytometry.

### Flow Cytometry

The expression of CD4, CD8, CD19, CD49b, γδ, F4/80, IL-6, and IL-17A was assessed in splenocytes or pulmonary cells by flow cytometry as previously reported (40, 41). The antibodies were conjugated to different fluorochromes (BD Biosciences, eBiosciences, San Diego, CA, USA, and Santa Cruz Biotechnologies, Santa Cruz, CA, USA). For intracellular cytokine staining, cells were previously permeabilized using PBS containing 1% FBS, 0.1% sodium azide, and 0.2% saponin. Data acquisition was performed using a FACSCanto II flow cytometer and FACSDiva software (BD Biosciences). Data were plotted and analyzed using FlowJo software (Tree Star, Ashland, OR, USA).

### Measurement of Cytokines in Lung Supernatant

The left lung (42) from uninfected and Pb18-infected mice were removed, weighed, homogenized in sterile PBS-containing protease inhibitor (Complete, Roche), and centrifuged (1,300 rpm, 10 min, 4°C). Supernatants were collected and stored at −20°C. The levels of IL-17A, IL-6, IL-23, TNF-α, and IFN-γ were measured by enzyme-linked immunosorbent assay (ELISA) according to manufacturers’ recommendations (BD Pharmingen, San Jose, CA, USA). The reading was held in eMax ELISA reader (Molecular Devices, Sunnyvale, CA, USA) at 450 nm.

### Recovery of Colony-Forming Units (CFUs)

The amount of viable yeast cells in the lungs from Pb18-infected mice (3–7 animals per group) was determined after 15 and 30 days postinfection (dpi). Briefly, lung lobules were aseptically collected, weighed, and homogenized using a sterile tissue grinder (IKA^®^-Werke, Deutschland, Germany). Each homogenate was diluted 1:10 in sterile PBS and plated (100 µL) on BHI agar supplemented with 5% FBS and gentamicin (100 µg/mL). Plates were incubated at 35–37°C for 7 days, and the amount of CFU per gram of tissue was calculated.

### Histopathology

Comparative histopathology was conducted with excised lung lobules from C57BL/6, IL-6^−/−^, IL-23^−/−^, and IL-17RA^−/−^ mice at 30 dpi. Tissue was fixed with 10% formalin for 24 h and embedded in paraffin. Next, lung sections (5 µm) were stained by standard procedures with hematoxylin and eosin for lesion analysis, Grocott methenamine silver stain for fungal cell labeling, or silver impregnation (Gomori method) to demonstrate reticulum fibers. Sections were examined with HLIMAGE++ 97 Application Western Vision Software using an optical microscope coupled to a digital camera. Granulomas were visually scored in mature or immature structures according to cellular and reticulin organization and distribution, as described before (43, 44). The percentage of mature granulomas and the granulomatous diameter were calculated. Reticulin measurement was performed in the halo surrounding the granulomas as previously described (45).

### Immunohistochemistry

Lungs from C57BL/6, IL-6^−/−^, IL-23^−/−^, and IL-17RA^−/−^ mice were immersed in OCT medium (Sakura Finetek), snap-frozen in liquid nitrogen, and stored at −80°C until analysis. Tissue sections (5 µm) were submitted to an immunohistochemical reaction. Briefly, slides were incubated with anti-mouse IgG as control or rabbit IgG anti-mouse inducible nitric oxide synthase (iNOS) enzyme (Santa Cruz Biotechnology, Santa Cruz, CA, USA) diluted 100 times in PBS 0.01% saponin. At the end, the reaction was followed by Mayer’s hematoxylin counterstaining. We measured immunostained areas using Image J software as previously described (41). Briefly, the range of positivity was defined using the IHC Tool box. Next, the images were converted to 8-bit, and the grayscale was converted to binary (black and white). The threshold was adjusted, and the labeled areas became the black portions. Finally, the percentage of stained area was analyzed.

### BMDMs Differentiation

Bone marrow cells isolated from femurs and tibias from 7-week-old C57BL/6, IL-6^−/−^, IL-23^−/−^, and IL-17RA^−/−^ naive mice were cultured (7 days, 37°C, 5% CO_2_) in RPMI 1640 medium supplemented with 20% FBS and 30% L-929 cell conditioned media, as previously described (46). After differentiation, cells were harvested and infected with *P. brasiliensis* (MOI of 0.04).

### Isolation of Naive T CD4^+^ Cells and Co-Culture with BMDMs

Naive T CD4^+^ cells were purified from C57BL/6 lymph nodes by using the CD4^+^ MicroBeads Isolation Kit (Miltenyi Biotec) in conjunction with an AutoMacs separator. Purified naive T cells were cultured for 5 days with *P. brasiliensis*-infected BMDMs from C57BL/6 or IL-6^−/−^ mice (MOI of 0.04). Next, the amount of T CD4^+^IL-17A^+^ was analyzed by flow cytometry.

### Adoptive Transfer

After adoptive transference of C57BL/6 BMDMs (1 × 10^6^, i.v.) into IL-6^−/−^, IL-23^−/−^, and IL-17RA^−/−^ animals, mice were infected with 1 × 10^6^ *P. brasiliensis* yeast cells, and the CFU was determined at day 30 as described above.

### Statistical Analysis

The differences observed between uninfected or Pb18-infected wild-type (WT) group (C57BL/6) and IL-6-, IL-17RA-, or IL-23-deficient mice were analyzed by applying the ANOVA test followed by multiple comparison using the Bonferroni method (GraphPad Prism, GraphPad Software version 5.0, San Diego, CA, USA). Values are expressed as mean ± SEM. All values were considered significant when *p* < 0.05.

## Results

### *P. brasiliensis* Infection Increases IL-17A Production

**Figure 1:**
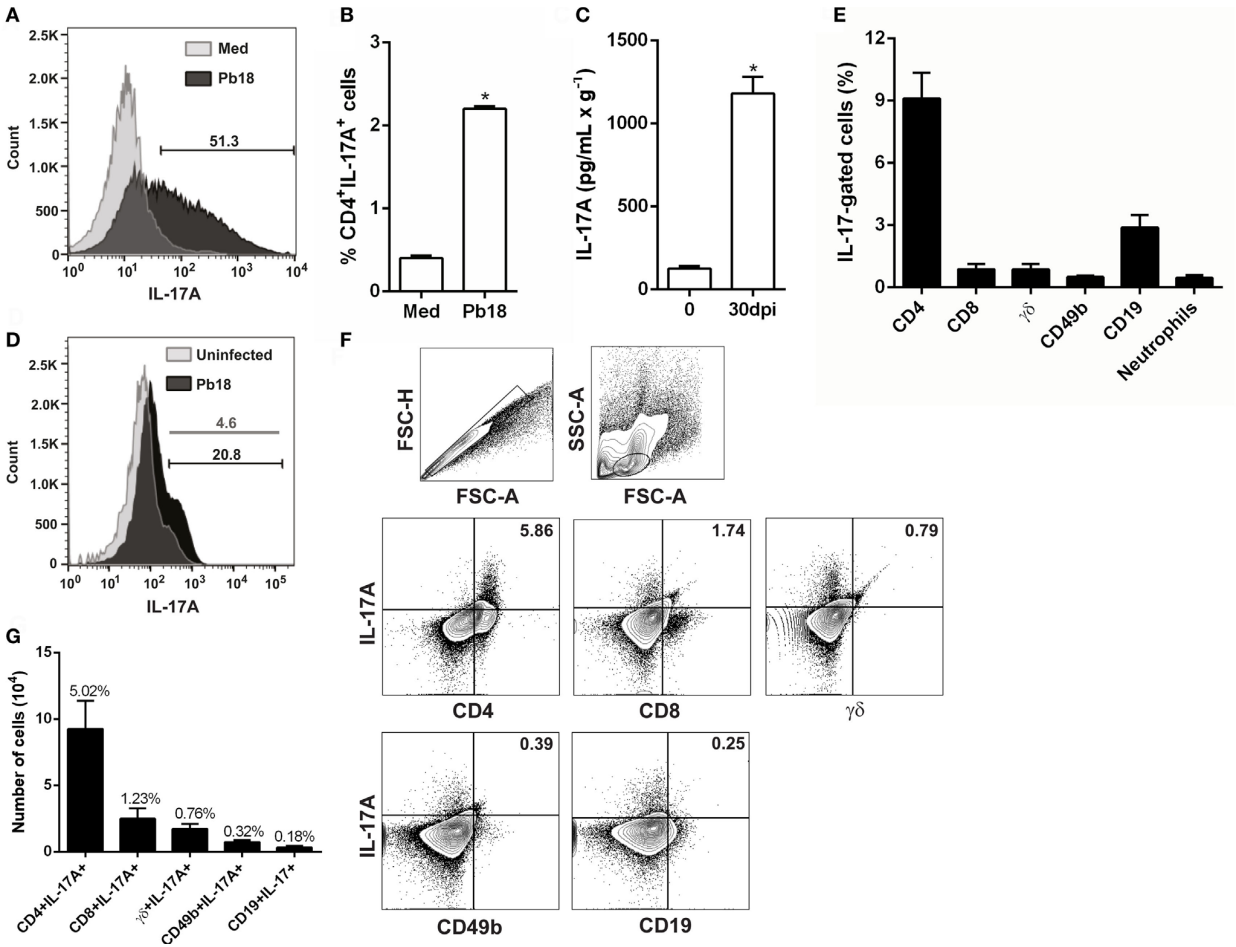
*Paracoccidioides brasiliensis* infection increases the *in vitro* and *in vivo* IL-17A production. **(A,B)** Splenocytes from C57BL/6 mice were isolated and cultivated (1 × 10^6^) in the presence of or not of viable virulent yeast strain 18 of *P. brasiliensis* (Pb18) during 5 days (multiplicity of infection of 1). **(A)** The frequency of IL-17A^+^-producing cells and **(B)** Th17 (CD4^+^IL-17A^+^) cells was determined by flow cytometry. **(C–E)** C57BL/6 mice were infected with 1 × 10^6^ Pb18 yeast cells (intravenously). **(C)** The level of IL-17A was measured by enzyme-linked immunosorbent assay in the whole lung homogenate after 30 days postinfection (dpi). **(D)** The frequency of IL-17A-producing cells was evaluated in the lungs from uninfected and infected (30 dpi) mice. **(E–G)** The frequency of natural killer (CD49b^+^IL-17A^+^), Tγδ (γδ^+^IL-17A^+^), B (CD19^+^IL-17A^+^), IL-17-producing CD8^+^ T (CD8^+^IL-17A^+^), and Th17 cells was assessed in the lungs after 30 dpi. **(E)** Cells were analyzed inside the IL-17-positive gate. **(F)** Lymphocytes were gated inside the singlet population. One representative mice is shown. **(G)** Absolute number (bars) and mean percentage (above bars) of each IL-17-producing cell population gated inside lymphocytes. Bars represent the mean ± SEM of 5 mice. *p < 0.05 compared to non-infected control. Similar results were obtained in three independent experiments.

To evaluate whether fungal cells modulate the IL-17A production, splenocytes from C57BL/6 mice were isolated, cultivated in the presence of or not of *P. brasiliensis*, and the frequency of IL-17A-producing cells was determined 5 days later. Our results showed that the stimulation of spleen cells with viable Pb18 increased the IL-17A expression (Figure 1A), and the frequency of IL-17A-producing CD4 T cells (5.5-fold, *p* < 0.05; Figure 1B) compared to medium alone. Next, to evaluate whether *P. brasiliensis* modulates the Th17 profile *in vivo*, C57BL/6 mice were infected with Pb18 yeast cells, and the IL-17A production was determined in the lungs at day 30 postinfection. As expected, we observed that Pb18-infected mice exhibited a significant increase (~9.5-fold) in IL-17A production compared to uninfected mice (Figure 1C). In addition, at 30 dpi, there was an increase in the number of IL-17A-producing cells (~4.5-fold; Figure 1D).

Despite the fact that most of the evidence describes IL-17A as a cytokine secreted by T cells, part of the IL-17A is produced by different cell populations, including innate immune cells (47). To determine the major source of IL-17A in the lungs during the experimental PCM, we evaluated the frequency and absolute number of Th17, Tc17, γδ^+^IL-17A^+^, CD49b^+^IL-17A^+^ [natural killer (NK)], and CD19^+^IL-17A^+^ (B cells). Initially, we excluded the doublets; delineated a gate comprising lymphocytes, monocytes, and neutrophils; and then a subgate in IL-17-positive cells, which allowed us to evaluate the percentage of each cell subtype (Figure 1E). The Th17 population was significantly increased in comparison with other IL-17-producing cells (*p* < 0.05; Figure 1E). For better and clear results, we next used a second gate strategy, which consisted of singlets selection in forward scatter height (FSC-H) against forward scatter area (FSC-A) (Figure 1F, upper left panel). Next, lymphocytes were gated inside the previous population in side scatter area (SSC-A) against FSC-A (Figure 1F, upper right panel), and all analyzed cell populations were gated inside the lymphocyte population. Our data clearly showed the predominance of IL-17A-producing CD4^+^ cells, both in percentage (~5%, Figures 1F,G) or absolute number (~9.2 × 10^4^ cells; Figure 1G), compared with other IL-17A^+^ cells subtypes: CD8 (~1.2%, ~2.5 × 10^4^ cells), γδ (~0.7%, ~1.7 × 10^4^ cells), NK (~0.3%, ~0.7 × 10^4^ cells), and B lymphocytes (~0.1%, ~0.3 × 10^4^ cells) (Figures 1F,G). After evaluating the number and frequency of IL-17-producing neutrophils, we could not see an increase of this cell subtype in the lungs from Pb18-infected mice (data not shown). These findings suggest that Th17 cells are the main source of IL-17A during *P. brasiliensis* infection.

### The Absence of IL-6 and IL-23 Impairs the Th17 Immune Response during Experimental *P. brasiliensis* Infection

**Figure 2:**
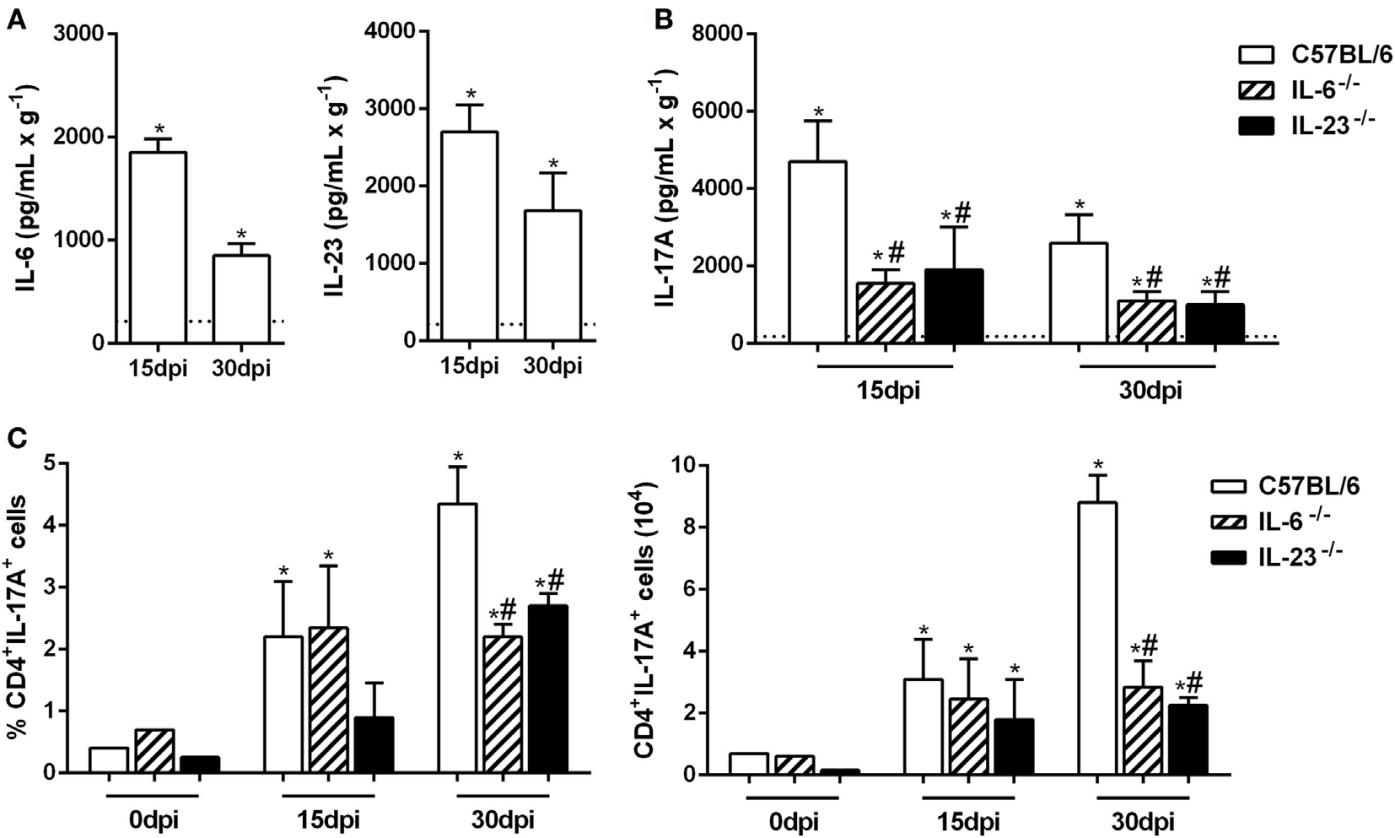
Virulent yeast strain 18 of *Paracoccidioides brasiliensis* (Pb18)-induced IL-6 and IL-23 production improves the establishment of the Th17 profile. **(A–C)** Male C57BL/6, IL-6^−/−^ and IL-23^−/−^ mice were infected with 1 × 10^6^ Pb18 yeast cells (intravenously). **(A)** IL-6 and IL-23 productions were quantified in the lung homogenate from uninfected and Pb18-infected C57BL/6 mice, at 15 and 30 days postinfection (dpi). **(B)** The IL-17A production was measured in the lung tissue from C57BL/6-, IL-6-, and IL-23-deficient mice. **(C)** The frequency and absolute number of Th17 (CD4^+^IL-17A^+^) cells were evaluated in the pulmonary tissue at 0, 15, and 30 dpi. The data represent the mean ± SEM of five mice and are representative of three independent experiments. Uninfected mice are represented by the dashed line. *p < 0.05 compared to uninfected mice. ^#^*p* < 0.05 compared to Pb18-infected C57BL/6 mice at the same time point.

The Th17 subset differentiation requires the presence of IL-6, and its maintenance is sustained by IL-23 (13, 14, 16). Thus, we evaluated the production of these cytokines in the lungs from naive and Pb18-infected mice at 15 and 30 dpi. Our data showed that at both time points, the IL-6 and IL-23 levels were significantly increased when compared to uninfected mice (Figure 2A). To confirm these findings, IL-6^−/−^ and IL-23^−/−^ mice were infected with *P. brasiliensis* yeast cells, and the IL-17A levels were evaluated in the pulmonary tissue after 15 and 30 days. We found that the deficiency of IL-6 or IL-23 impaired the IL-17A-production, which was ~2.5 times smaller than C57BL/6 mice at the same period of infection (*p* < 0.05; Figure 2B). Moreover, when compared to WT mice, we found that the frequency and absolute number of IL-17A-producing CD4^+^ T cells were decreased (*p* < 0.05) in the lung tissue from IL-6^−/−^ and IL-23^−/−^ mice at 30 dpi (Figure 2C). These data establish the importance of IL-6 and IL-23 in promoting the late Th17-immune response during the *P. brasiliensis* experimental infection.

### Th17-Associated Cytokines Contribute to Compact Granuloma Formation during the Experimental *P. brasiliensis* Infection

**Figure 3:**
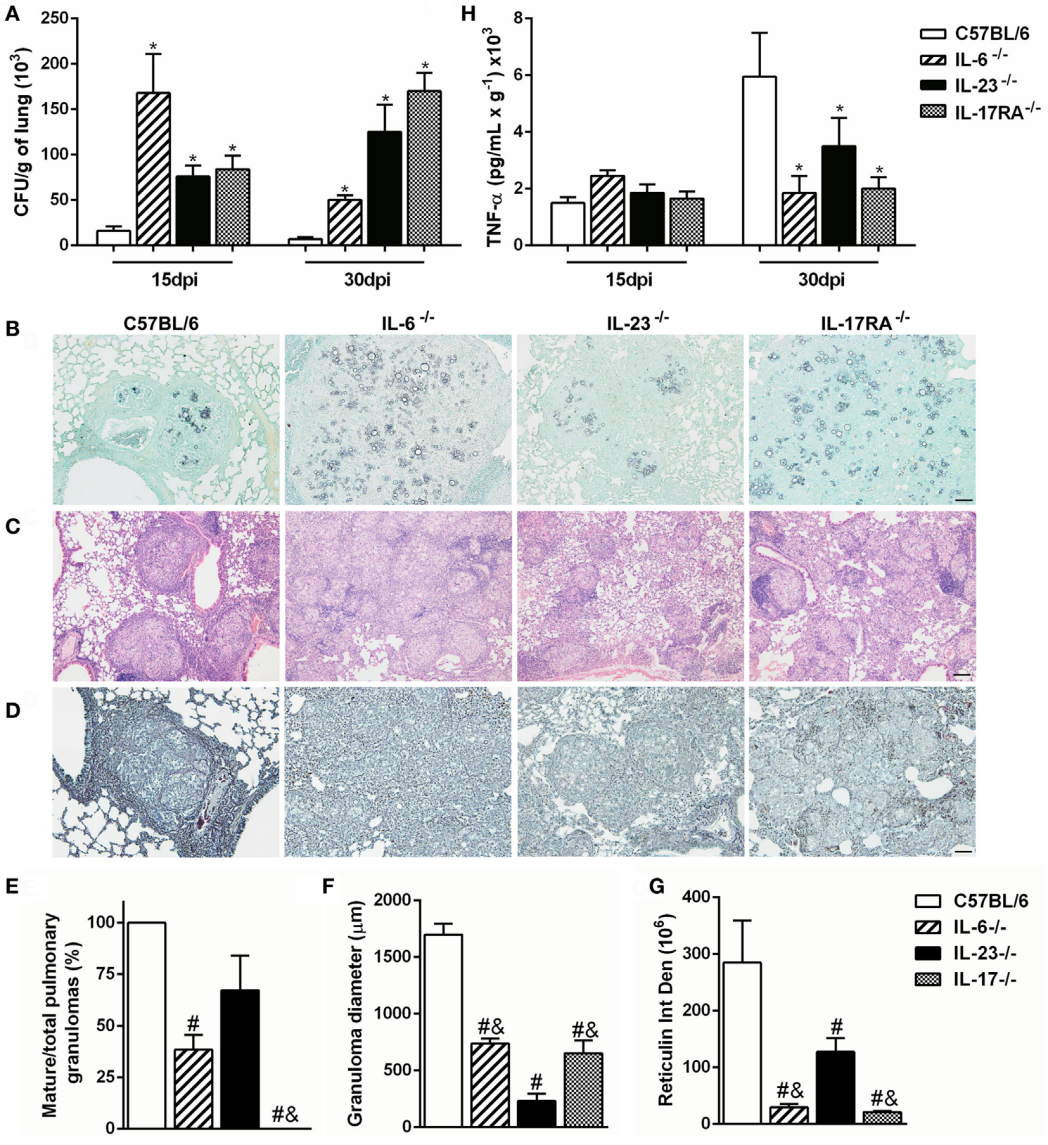
The Th17-associated cytokines contribute to development and organization of granuloma during experimental *Paracoccidioides brasiliensis* infection. **(A–E)** C57BL/6, IL-6^−/−^, IL-23^−/−^, and IL-17 receptor A (IL-17RA)^−/−^ mice were infected with virulent yeast strain 18 of *P. brasiliensis* (Pb18) (1 × 10^6^ cells, intravenously). **(A)** The number of colony-forming units (CFUs) was determined in the pulmonary tissue after 15 and 30 days postinfection (dpi). **(B–D)** Lung sections of infected mice were stained with **(B)** Grocott methenamine silver, **(C)** hematoxylin and eosin, or **(D)** silver impregnation (Gomori method) at 30 dpi and analyzed by light microscopy. Scale bar: 50 µm **(B,D)** and 100 µm **(C)**. **(E)** The percentage of mature granulomas and **(F)** diameter of mature and immature granulomas were calculated. **(G)** Integrated density of reticulin present in the halo surrounding the mature granulomas. **(H)** The tumor necrosis factor (TNF)-α production was measured in the lung homogenate from Pb18-infected mice at 15 and 30 dpi. Similar results were obtained in three independent experiments. Data represent the mean ± SEM of five mice. Uninfected mice are represented by the dashed line. *p < 0.05 compared to uninfected mice. ^#^*p* < 0.05 compared to Pb18-infected C57BL/6 mice at the same time point. ^&^*p* < 0.05 compared to Pb18-infected IL-23^−/−^ mice at the same time point.

To verify whether *P. brasiliensis*-induced Th17-associated cytokines are important to control the fungal growth and pulmonary infection, we analyzed the amount of CFU and the histopathology of lung tissue from Pb18-infected IL-6-, IL-23-, and IL-17RA-deficient mice. Our data revealed that all deficient mice showed impaired fungal control at both 15 and 30 days after *P. brasiliensis* inoculation when compared to C57BL/6 mice (*p* < 0.05; Figure 3A). Likewise, the Grocott stain at 30 dpi corroborated a higher fungal load in the lungs of all deficient mice when compared to infected C57BL/6 mice (Figure 3B), suggesting that Th17-associated cytokines, as IL-6 and IL-23, are important to control the fungal growth during the experimental PCM in mice. To better assess the inflammatory process developed during *P. brasiliensis* infection, we analyzed the histology of pulmonary tissue at 30 dpi. The C57BL/6 mice showed bigger and mature granulomas characterized by epithelioid and giant cells surrounded by a peripheral mononuclear layer (Figures 3C,E,F). In contrast, most of the granulomas from IL-6^−/−^ and IL-17RA^−/−^ mice exhibited an intermediate size and immature profile with few lymphocytes forming the peripheral ring, associated with a diffuse inflammatory process (Figures 3C,E,F). Specifically, the IL-23 deficiency induced the formation of numerous well-formed granulomas, but with a smaller diameter compared to all Pb18-infected groups (Figures 3C,E,F).

Reticulin fibers are composed of type III collagen; besides very delicate and fine, they form a firm layer linking the connective to the surrounding tissue, being considered a main element of alveolar organization (48). We observed a well-organized and dense reticulin ring contouring the granulomas in the lungs from Pb18-infected C57BL/6 mice (Figures 3D,G). In addition, the diminished number of compact granulomas in IL-6^−/−^ and IL-17RA^−/−^ pulmonary tissue was associated with a defective reticulin halo accumulation and formation (Figures 3D,G). Moreover, a tiny reticulin layer surrounded the small mature and immature granulomas in the lungs from IL-23-deficient mice at 30 days post-Pb18 infection (Figures 3D,G).

Since TNF-α is important to the granuloma formation (36, 37), we also evaluated the expression of this cytokine in the lung tissue from Pb18-infected C57BL/6, IL-6^−/−^, IL-23^−/−^, and IL-17RA^−/−^ mice. Our data showed that *P. brasiliensis* infection increased the TNF-α production at both evaluated time points (Figure 3H). There were no differences in TNF-α levels in the various noninfected knock-out mice (data not shown). At 15 dpi, all groups showed similar TNF-α production. However, when compared to C57BL/6 mice, the levels of TNF-α were reduced in all deficient mice at 30 dpi (*p* < 0.05; Figure 3H). These results suggest that IL-6, IL-17A, and in less extent, IL-23 modulate the formation of organized and compact granulomas during the experimental *P. brasiliensis* infection.

### Th17-Associated Cytokines Promote IFN-γ Production and iNOS Expression during the Experimental *P. brasiliensis* Infection

**Figure 4:**
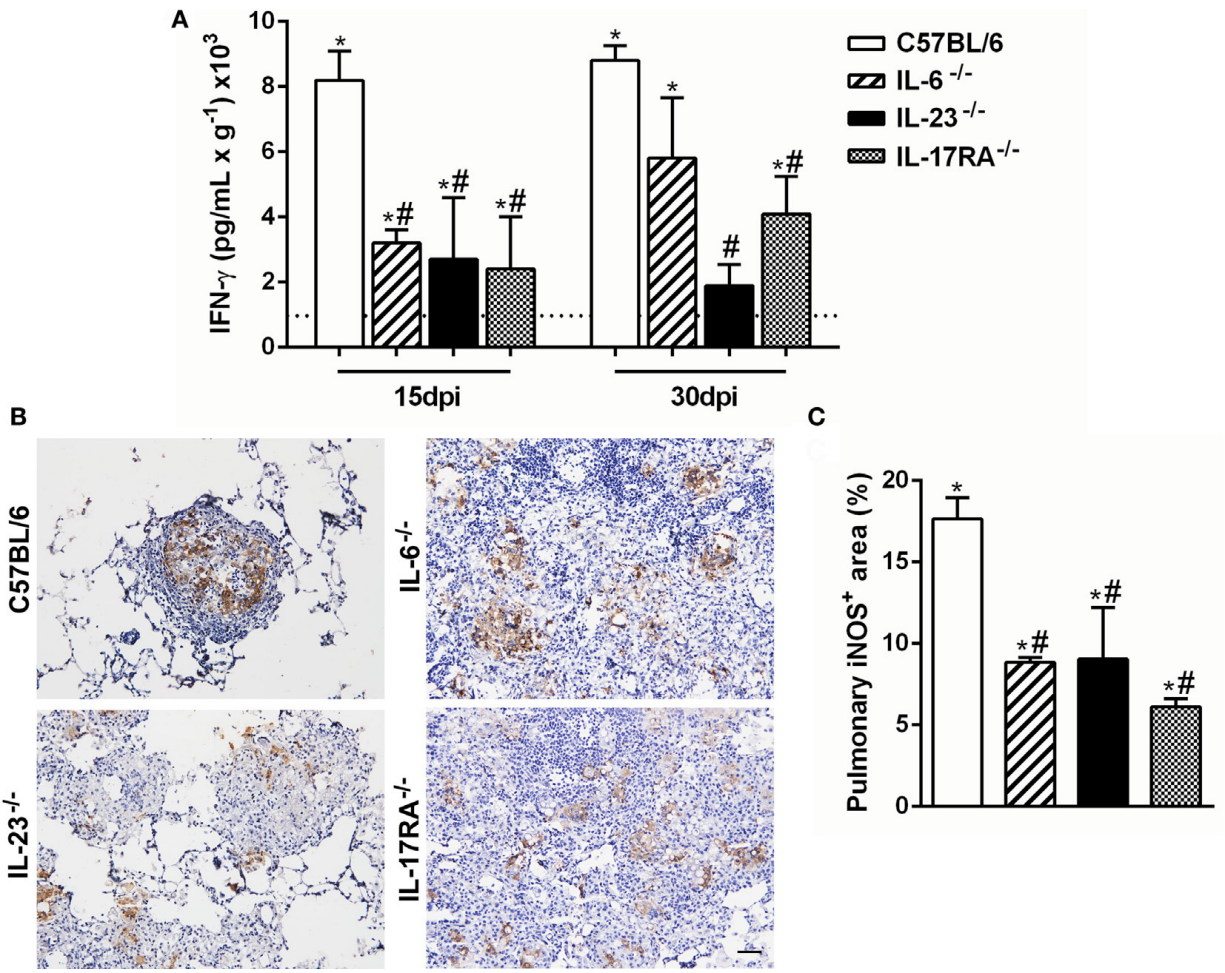
Th17-associated cytokines regulate the IFN-γ production and inducible nitric oxide synthase enzyme (iNOS) expression during the experiment of virulent yeast strain 18 of *Paracoccidioides brasiliensis* (Pb18) infection. **(A,B)** C57BL/6, IL-6^−/−^, IL-23^−/−^, and IL-17 receptor A (IL-17RA)^−/−^ mice were infected (intravenously) with 1 × 10^6^ Pb18 yeast cells. **(A)** IFN-γ levels were measured by enzyme-linked immunosorbent assay in the lung homogenate at 15 and 30 days postinfection (dpi). **(B)** Immunohistochemistry was performed in lung tissue to analyze the iNOS expression at 30 dpi. **(C)** The percentage of the pulmonary area containing iNOS^+^ cells is indicated. Lung sections were stained for iNOS at 30 dpi using immunohistochemistry. Scale bar: 50 µm. Similar results were obtained in three independent experiments. Data represent the mean ± SEM of five mice. Uninfected mice are represented by the dashed line. *p < 0.05 compared to uninfected mice. ^#^*p* < 0.05 compared to Pb18-infected C57BL/6 mice at the same time point.

Several studies have demonstrated that the Th1-type cytokine IFN-γ is implicated in resistance during *P. brasiliensis* infection, inducing cell migration and activation (37, 49). Then, we next evaluated the importance of IL-6, IL-17A, and IL-23 in modulating the IFN-γ-production during the experimental PCM. All naive knock-out mice showed similar IFN- γ levels (data not shown), and these levels were increased after the *P. brasiliensis* infection (Figure 4A). At both evaluated time points, 15 and 30 dpi, all deficient mice showed a reduction of IFN-γ levels in the pulmonary tissue in comparison to C57BL/6 group (Figure 4A). These findings suggest that Th17-associated cytokines are involved in the IFN-γ production during the experimental PCM in mice.

It was previously demonstrated that IFN-γ is able to induce fungicidal activity in macrophages through nitric oxide (NO) production (50, 51), which is indirectly measured by iNOS expression. At 15 dpi, there was increased iNOS expression inside the granulomatous structure from C57BL/6 mice, in comparison with all analyzed deficient mice at the same time point (Figures 4B,C).

Together, these results suggest that the deficiency of Th17-associated cytokines (IL-6 and IL-23) modulates the IFN-γ production and iNOS expression and dampens the fungal death during an experimental PCM model.

### Adoptive Transfer of F4/80^+^ Cells Restores Resistance to *P. brasiliensis* Infection in IL-6- and IL-17RA-Deficient Mice

**Figure 5:**
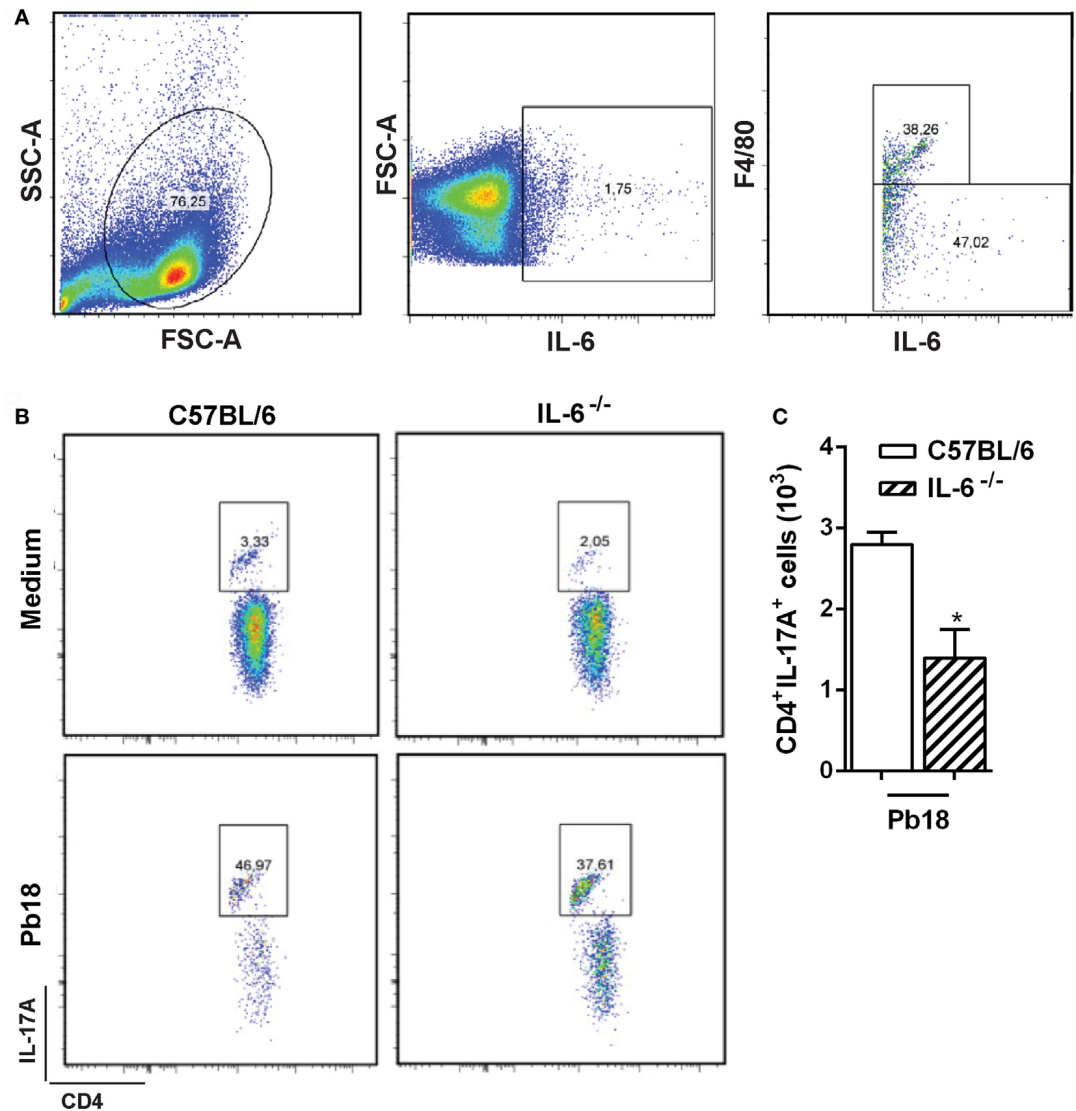
Macrophage-derived IL-6 improves Th17 differentiation during virulent yeast strain 18 of *Paracoccidioides brasiliensis* (Pb18) experimental infection. **(A)** Splenocytes from C57BL/6 mice were isolated and cultivated in the presence of or not of viable Pb18 yeasts cells during 5 days, and the frequency of IL-6-producing F4/80^+^ macrophages was analyzed by flow cytometry. **(B,C)** Bone marrow-derived macrophages were differentiated from C57BL/6 and IL-6^−/−^ mice and co-cultured with naive T CD4^+^ cells from C57BL/6 wild-type mice in the presence of or not of Pb18 yeast cells (multiplicity of infection of 0.04) during 5 days. Next, the frequency **(B)** and absolute number **(C)** of Th17 (CD4^+^IL-17A^+^) cells were analyzed by flow cytometry. Similar results were obtained in three independent experiments. Data represent the mean ± SEM of five mice. *p < 0.05 compared with C57BL/6 mice.

We first showed that ~38% of F4/80^+^ cells (macrophages) from splenocytes cultured with Pb18 yeast cells are able to produce IL-6 (Figure 5A). Gate strategy consisted of doublets exclusion in FSC-H against FSC-A (data not shown). Next, monocytes were gated in SSC-A against FSC-A (Figure 5A, left panel), and then the IL-6-positive cells were selected (Figure 5A, middle panel). Finally, the IL-6^+^F4/80^+^ population was delimited (Figure 5A, right panel). We next evaluated the role of IL-6 in Th17 differentiation during Pb18 infection. The BMDMs from C57BL/6 and IL-6^−/−^ mice were co-cultured with naive CD4^+^ lymphocytes isolated from C57BL/6 WT mice, in the presence of or not of Pb18 yeast cells. We observed that the frequency and absolute number of Th17 cells induced by WT BMDMs were significantly higher than that of IL-6^−/−^ BMDMs (*p* < 0.05; Figures 5B,C, respectively). Overall, these data suggest that the IL-6 produced by F4/80^+^ macrophages induces Th17 cells during the *P. brasiliensis* infection.

**Figure 6:**
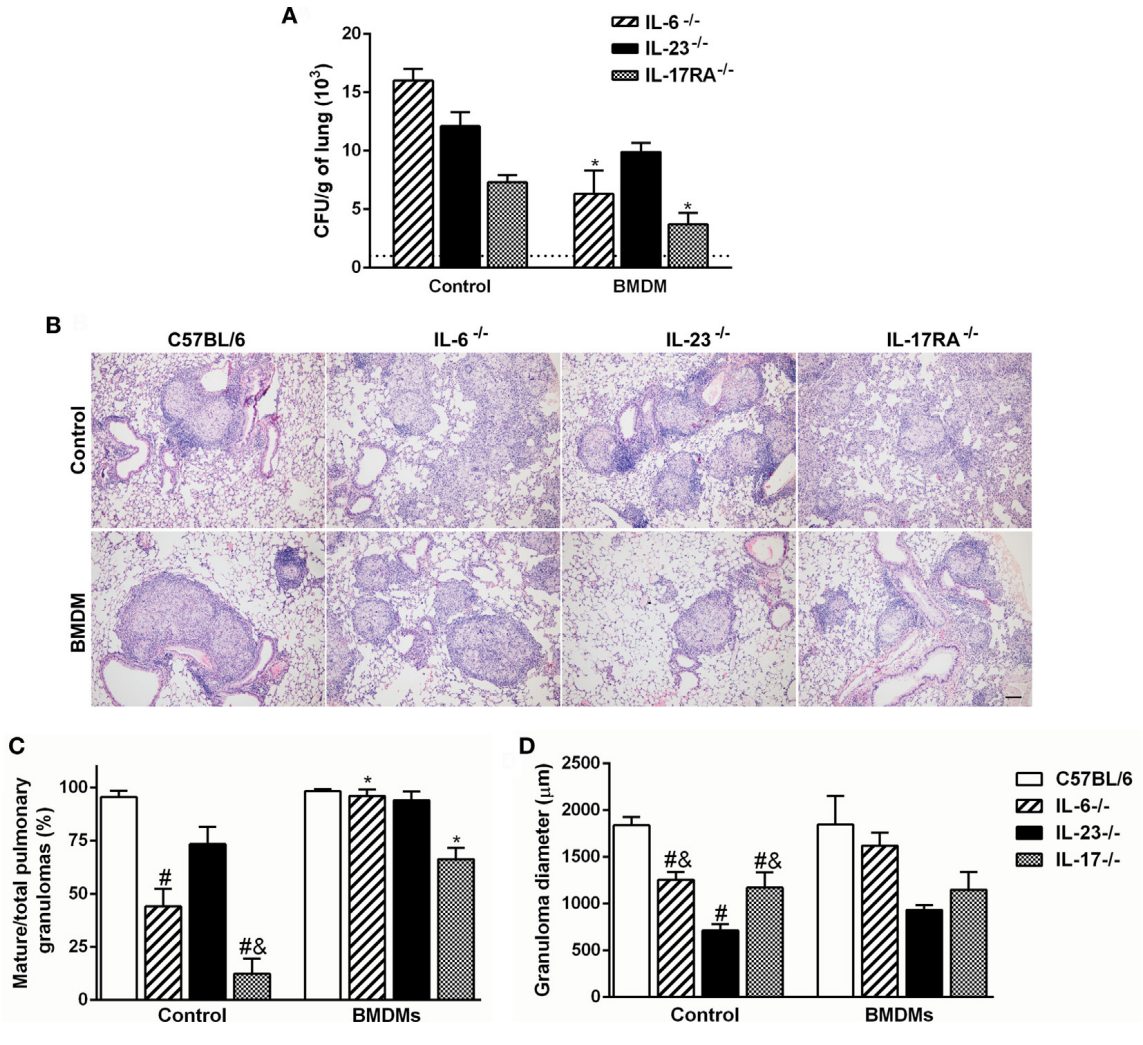
Adoptive transfer of wild-type F4/80^+^ macrophages restores the granuloma formation and resistance during *Paracoccidioides brasiliensis* infection in susceptible IL-6- and IL-17 receptor A (IL-17RA)-deficient mice. **(A,B)** Macrophages (1 × 10^6^), previously differentiated *in vitro* from C57BL/6 bone marrow-derived macrophages (BMDMs), were adoptively transferred to C57BL/6, IL-6^−/−^, IL-23^−/−^, and IL-17RA^−/−^ mice, followed by the intravenous infection with 1 × 10^6^ virulent yeast strain 18 of *P. brasiliensis* (Pb18). **(A)** The number of colony-forming unit (CFU) was determined in the pulmonary tissue at day 30 postinfection. Dashed line represents the amount of CFU in C57BL/6 mice. **(B)** Lung sections of Pb18-infected mice, transferred or not with wild-type BMDMs, were stained with hematoxylin and eosin. **(C)** The percentage of mature granulomas and **(D)** diameter of mature and immature granulomas was calculated. Scale bar: 100 µm. Similar results were obtained in three independent experiments. Data represent the mean ± SEM of five mice. ^#^*p* < 0.05 compared to Pb18-infected C57BL/6 mice without BMDMs transfer (control). ^&^*p* < 0.05 compared to control IL-23^−/−^. *p < 0.05 compared with same genotypes without transfer.

To verify the relevance of Th17-associated cytokines over the macrophage fungicidal activity during *P. brasiliensis* infection, BMDMs from C57BL/6 mice were transferred to IL-6-, IL-23-, and IL-17RA-deficient mice, and the fungal burden was evaluated after 30 dpi. To confirm that transferred cells arrive in the lungs, we labeled BMDMs from GFP^+^ mice with F4/80, and at day 7 after transference, we tracked these cells. We verified a frequency of ~7.3% of F4/80^+^GFP^+^ cells in the pulmonary tissue (data not shown). The IL-6^−/−^ and IL-17RA^−/−^ transferred mice showed diminished number of Pb18 yeast cells in comparison with same genotypes without transfer (*p* < 0.05; Figure 6A). However, the adoptive transfer of BMDMs did not change the fungal burden in IL-23^−/−^ mice (Figure 6A).

Since resistance to *P. brasiliensis* infection is characterized by the presence of mature granulomas, we evaluated the influence of macrophages in the granuloma formation. Corroborating our previous findings (Figure 3C), the Pb18-infected IL-6- and IL-17RA-deficient mice showed diminished capacity to mount organized granulomas at 30 dpi when compared to C57BL/6 group (Figures 6B,C). Surprisingly, the adoptive transfer of WT BMDMs restored the ability of IL-6^−/−^ and IL-17RA^−/−^ mice to form mature granulomas in the lung tissue (Figures 6B,C). We could not observe histological differences in Pb18-infected IL-23-deficient mice transferred or not with C57BL/6 BMDMs, which continued showing small well-formed granulomas in the lungs after transference (Figures 6B,D).

Taken together, our data suggest that Th17-associated cytokines are involved in the modulation of immune response and granuloma formation during experimental PCM.

## Discussion

The generation of a protective immune response is essential for human resistance against infectious diseases, including the systemic mycosis caused by *P. brasiliensis*. If the host innate immune response is unable to suppress the infection foci, it is followed by cell-mediated immunity (52). Several studies have demonstrated that the severe PCM occurs due to the inability to develop an effective Th1 response and, therefore, unsuitable for the formation of dense granulomas, while Th2 response is inefficient to contain the spread of infection (53). Since Th17 discovery, these cells have been associated with immune response to fungal diseases and considered one of the main mechanisms that cause resistance to mycosis (23).

In fact, the production of IL-17A is instrumental for protective response in an experimental model of *P. carinii* infection (26). In addition, Th17-associated cytokines improve the host control against *C. albicans* and *Aspergillus fumigatus*, leading to the migration of potentially fungicide neutrophils (54). Moreover, the presence of IL-17A-producing cells in lesions of PCM patients was demonstrated, in an attempt to increase the host immune defenses against fungal cells (38, 55). In agreement, here, we demonstrated the increased expression of Th17-associated cytokines (IL-6, IL-23, and IL-17A) in the lung tissue after the *P. brasiliensis* infection in mice.

Besides Th17, IL-17A is synthesized by NK cells, γδ T cells, neutrophils, and innate lymphoid cells, in a more rapid way than T cells due to the constitutive expression of the Th17-associated transcription factor RORγt in all these cells subtypes (47). During experimental PCM, our results showed that Th17 lymphocytes are the main source of IL-17A in the lungs, followed by CD8^+^ cells. The presence of Tc17s in the lungs from *P. brasiliensis-*infected mice or in cutaneous and mucosal lesions from PCM patients was showed before (30, 38). Moreover, Tc17 differentiation of naive T lymphocytes was demonstrated *in vitro* after Pb18 infection (31). During *A. fumigatus* infection, neutrophils were positive for intracellular IL-17A expression and also produced this cytokine in a Dectin-1- and IL-23-dependent manner (56). However, the *P. brasiliensis* infection did not modulate the IL-17-producing neutrophils in the lungs. Moreover, we found that IL-6 is important to the protective response during the infection. Similarly, previous work demonstrated that IL-6 increases murine resistance to *P. brasiliensis* yeast cells (57). Previous work highlighted the critical roles of IL-1 during early Th17 differentiation and expansion/maintenance (11). Our group partially elucidated the mechanisms modulated by IL-1 during PCM (58). We have found that *P. brasiliensis* promoted a NLRP3 inflammasome-dependent caspase-1 activation to release the bioactive IL-1β, and IL-1R1^−/−^ mice displayed a slightly increased survival rate to infection compared with the C57BL/6 mice. Moreover, significantly higher amounts of the fungus were recovered at 30 dpi in the lungs of the IL-1R1-deficient mice, which were contained in organized and compact granulomas (58). These data corroborate the idea that the absence of cytokines associated with Th17 differentiation may increase the susceptibility to experimental PCM.

Granulomas are formed as a consequence of chronic antigen persistence, and their formation involves the interaction between the antigenic organism and host immune cells (48). During primary tuberculosis, IL-17-producing cells are induced, leading to IL-17A synthesis, which is a potent inflammatory cytokine capable of increase chemokines expression that promotes cell recruitment and granuloma organization (59). Similarly, it is possible that IL-17A is leading to cell migration during experimental PCM, contributing to granuloma formation. Moreover, we showed that the Th17-associated cytokine IL-6 constitutes an essential molecule involved in granuloma development during the *P. brasiliensis* infection. Here, we established for the first time the importance of IL-6 and IL-23 for the Th17 induction/expansion during experimental PCM. According to our results, these cytokines play a critical role in the protection against *P. brasiliensis* yeast cells through the induction of mature granuloma formation and control of fungal growth.

The resistance observed in some PCM patients is dependent on cellular activities mediated by IFN-γ and TNF-α. An efficient host immune response and a potent fungicidal activity against *P. brasiliensis* are determined by the synergistic effect between these two cytokines (37, 49). Several studies have demonstrated that Th17 cells can turn into IFN-γ-expressing T cells (60–62). IFN-γ stimulates *P. brasiliensis*-infected macrophages to secrete TNF-α, required for the development and persistence of well-formed granulomas (36) and is important for host resistance against fungal cells (63). Our data showed a diminished IFN-γ and TNF-α production in the pulmonary tissue from IL-6^−/−^, IL-23^−/−^, and IL-17RA^−/−^ mice when compared to C57BL/6 group. The lower production of IFN-γ and TNF-α correlated with impaired granuloma formation in the lung tissue after *P. brasiliensis* infection. In addition, IFN-γ induces inflammatory cells to produce NO, which plays a well-documented role in fungal clearance (64). Besides IFN-γ, the NO production in chronic inflammation is supported by IL-17A (65, 66). This statement justify the decreased iNOS expression inside the granulomatous structures in the lung tissue from *P. brasiliensis*-infected IL-6-, IL-23-, and IL-17RA-deficient mice, associated with increased fungal load.

It is known that IL-17A increases the macrophage activity and survival (67). However, the importance of macrophages in Th17 cell responses is still poorly understood. An inflammatory stimulus is able to increase the expression of IL-17A receptors in macrophages both *in vitro* and *in vivo* (68). Interestingly, mycobacterial infection induces a new type of macrophage population, different from M1/M2 macrophages, that downregulates T cell production of both Th1- and Th2-type cytokines but markedly increases the production of IL-17A and IL-22 through upregulation of Th17 cell expansion, *via* IL-6 and TGF-β, but not IL-21 and IL-23 (69). Similarly, we found that *P. brasiliensis* experimental infection induced IL-6 synthesis even in the early infection, and this cytokine is essential to induce a Th17 immune profile. In corroboration, we showed that IL-6 deficiency in macrophages is associated with abrogated Th17 cell generation, diminished fungal load in the lungs, and absence of organized granulomas. The adoptive transfer of IL-6-competent macrophages restored the resistance in *P. brasiliensis*-infected IL-6- and IL-17RA-deficient mice, but did not change the granuloma formation and fungal clearance in IL-23^−/−^ group.

Taken together, our data suggest that macrophage-produced IL-6 and IL-23 are essential to induce a Th17 immune profile, which positively regulates the TNF-α, IFN-γ, and iNOS expression, contributing to mature granuloma formation and consequent better control of *P. brasiliensis* experimental infection.

## Ethics Statement

Experiments were conducted according to the ethical principles of animal research adopted by the Brazilian College of Animal Experimentation (COBEA) and approved by the Ethical Commission in Animal Research (protocol 095/2010).

## Author Contributions

FT, FR, DC, NK-C, CS, and CM performed the *in vitro* and *in vivo* experiments. FT, FR, DC, NK-C, and JS designed protocols. FT, FR, and DC conducted data analysis. FT, FR, DC, NK-C, CS, CM, and JS provided scientific input. JS supervised the project. FT reviewed the literature and wrote the manuscript. DC, NK-C, and JS provided comments and corrections of the manuscript.

## Conflict of Interest Statement

The authors declare that the research was conducted in the absence of any commercial or financial relationships that could be construed as a potential conflict of interest.
